# Prime Editing for Crop Improvement: A Systematic Review of Optimization Strategies and Advanced Applications

**DOI:** 10.3390/genes16080965

**Published:** 2025-08-16

**Authors:** Shuangrui Tian, Lan Yao, Yuhong Zhang, Xiaoyu Rao, Hongliang Zhu

**Affiliations:** 1 College of Food Science & Nutritional Engineering, China Agricultural University, Beijing 100083, China; tian-sr@cau.edu.cn (S.T.); donnayao@cau.edu.cn (L.Y.); 2022306100502@cau.edu.cn (Y.Z.); abu145914@cau.edu.cn (X.R.); 2 Sichuan Advanced Agricultural & Industrial Institute, China Agricultural University, Chengdu 611430, China

**Keywords:** prime editing, genome editing, crop improvement, systematic review, editing efficiency, optimization strategy, plant biotechnology

## Abstract

Prime editing (*PE*), a novel “search-and-replace” genome editing technology, demonstrates significant potential for crop genetic improvement due to its precision and versatility. However, since its initial application in plants, *PE* technology has consistently faced challenges of low and variable editing efficiency, representing a major bottleneck hindering its broader application. Therefore, this study conducted a systematic review following the *PRISMA* 2020 guidelines. We systematically searched databases—Web of Science, PubMed, and Google Scholar—for studies published up to June 2025 focusing on enhancing *PE* performance in crops. After a rigorous screening process, 38 eligible primary research articles were ultimately included for comprehensive analysis. Our analysis revealed that early *PE* systems such as *PE2* could perform diverse edits, including all 12 base substitutions and small insertions or deletions *(indels)*, but their efficiency was highly variable across species, targets, and edit types. To overcome this bottleneck, researchers developed four major optimization strategies: (1) engineering core components such as *Cas9*, reverse transcriptase (*RT*), and editor architecture; (2) enhancing expression and delivery via optimized promoters and vectors; (3) improving reaction processes by modulating *DNA* repair pathways or external conditions; and (4) enriching edited events through selectable or visual markers. These advancements broadened *PE*’s targeting scope with novel *Cas9* variants and enabled complex, kilobase-scale *DNA* insertions and rearrangements. The application of *PE* technology in plants has evolved from basic functional validation, through systematic optimization for enhanced efficiency, to advanced stages of functional expansion. This review charts this trajectory and clarifies the key strategies driving these advancements. We posit that future breakthroughs will increasingly depend on synergistically integrating these strategies to enable the efficient, precise, and predictable application of *PE* technology across diverse crops and complex breeding objectives. This study provides an important theoretical framework and practical guidance for subsequent research and application in this field.

## 1. Introduction

In the 21st century, human society faces the dual pressures of global climate change and continuous population growth, making ensuring global food security an unprecedented challenge. To address this challenge, developing crop varieties with high yield, superior quality, and enhanced resilience (e.g., disease resistance, drought tolerance, salinity tolerance) constitutes a core task of modern agriculture. Traditional hybrid breeding methods once achieved the glory of the “Green Revolution”. However, these traditional methods are inadequate to meet the enormous future demand for food. Their limitations include long breeding cycles, a heavy reliance on existing genetic variation, and difficulty in achieving precise and efficient trait improvement [1]. The advent of gene editing technologies, such as the *CRISPR/Cas9* system, has enabled targeted modification of genetic information. These tools work by creating *DNA* double-strand breaks (*DSBs*) at specific genomic sites, which, in turn, activate the cell’s intrinsic repair mechanisms [2,3,4]. However, *CRISPR/Cas9* technology primarily relies on the error-prone non-homologous end joining (*NHEJ*) repair pathway within cells, often resulting in unpredictable random *indels*. Conversely, the homology-directed repair (*HDR*) pathway, capable of achieving precise sequence replacement, exhibits extremely low efficiency in the vast majority of plant cell types [5]. To circumvent the risks associated with *DSBs*, researchers developed base editing (*BE*) technology, which operates without cleaving the *DNA* double helix. Nevertheless, *BE* technology has significant limitations. Its application is restricted to four types of base transitions (*C→T*, *G→A*, *A→G*, *T→C*) and is unable to perform base transversions or small *indels* [6,7]. Crucially, its specificity is also a major concern due to the ‘bystander effect,’ where unintended, similar bases within a defined editing window are converted alongside the target base, resulting in unwanted mutations. Consequently, developing a more versatile, precise, and safer gene editing tool is crucial for propelling crop genetic improvement into a new era.

To overcome the limitations of the technologies above, an innovative gene editing technology named prime editing (*PE*) emerged [8]. The core of the *PE* system is a fusion protein comprising a *Cas9* nickase (*nCas9*, which cleaves only a single *DNA* strand) and reverse transcriptase (*RT*), guided by a specially engineered *PE* guide *RNA* (*pegRNA*). Guided by the *pegRNA*, this fusion protein generates a single-strand nick at the target *DNA* site. The exposed 3′-hydroxyl group at the nick serves as a primer for in situ reverse transcription, using the template sequence carried by the *pegRNA* itself as a blueprint. This “search-and-replace” mechanism can, in principle, achieve all 12 types of single-base substitutions and precise multi-base *indels*. Critically, it accomplishes this without generating *DSBs* or requiring an exogenous repair template. Owing to its high versatility, precision, and safety, *PE* technology provides an unprecedentedly powerful tool for performing fine, surgical operations on the genome [8].

Shortly after its seminal publication, *PE* technology was rapidly applied in the plant field. Researchers successfully demonstrated its capability for diverse precise edits in important crops such as rice and wheat [9], confirming its significant potential for crop genetic improvement [10]. Subsequently, its application scope quickly expanded to a wider range of plant species, including maize, tomato, and tobacco [11,12]. However, a core challenge has emerged with deeper application. Despite its powerful functionality, *PE* efficiency in plants is often low and highly unpredictable [13,14]. This issue presents a practical bottleneck that hinders its widespread adoption. This instability manifests at multiple levels. First, there are substantial differences between species. For instance, while the *PE* system can achieve desirable editing efficiency in rice, its efficacy is often minimal in important economic crops like tomato and legumes [15,16]. Second, even within the same species (e.g., rice), efficiency varies drastically depending on the target gene. Editing efficiency targeting the *OsCDC48* gene can be as high as 29.17%, whereas no successful events were detected for editing the *OsACC1* gene [17]. Finally, efficiency is also critically dependent on the edit type and *pegRNA* design. Studies found that for performing the same base substitution at the same gene locus, simply using four different *pegRNA*s resulted in efficiencies fluctuating wildly within a broad range of 0.0% to 14.6% [14]. This profound variability in performance across different species, target sites, and experimental designs underscores the core challenge of “unstable efficiency” for the *PE* system in plants. Therefore, a systematic review of existing optimization strategies is paramount. Such an analysis is essential for advancing *PE* technology toward practical field applications and realizing the goal of precise crop breeding.

Facing the core challenge of unstable *PE* efficiency in plants, researchers globally have systematically driven the evolution of this technology from two interrelated perspectives. On the one hand, efforts focus on enhancing its core efficacy. This involves addressing the bottleneck of low base editing efficiency through multiple strategies, including engineering the editor protein and *pegRNA*, optimizing expression and delivery systems, modulating the endogenous cellular environment, and innovating screening methods. On the other hand, building upon improved efficiency, there is a continuous push to expand the capability boundaries of *PE* technology to meet more advanced breeding needs. This includes breaking the constraints of protospacer adjacent motif (*PAM*) sequences to broaden the targeting scope and developing complex editing systems capable of achieving long *DNA* fragment insertions, deletions, and even chromosome-level rearrangements that were previously difficult to attain. This review follows the *PRISMA* guidelines to provide a comprehensive analysis of research progress. We systematically present the evolutionary trajectory of *PE* technology across two key dimensions: the enhancement of its efficiency and the expansion of its capabilities. Thereby, it seeks to provide a theoretical foundation and practical guidance for the in-depth research and broad application of this technology in future crop genetic improvement.

## 2. Methods

### 2.1. Study Selection

We adhered to best practice guidelines as detailed by the *PRISMA* framework for systematic reviews, specifically focusing on the efficiency enhancement of *PE* technology in crop plants. This tool is recognized as a method for improving both the reporting and conduct of systematic reviews, including those focused on specific research questions [18].

To identify eligible studies, we systematically searched three major databases: Web of Science, PubMed, and Google Scholar (covering the period from January 2019 to June 2025). Our search employed Boolean logic: (“prime editing”) AND (plant OR crop OR rice OR “*Oryza sativa*” OR wheat OR “*Triticum aestivum*” OR maize OR *Zea* OR tomato OR “*Solanum lycopersicum*” OR “*Lycopersicon esculentum*” OR potato OR “*Solanum tuberosum*” OR Tobacco OR “*Nicotiana tabacum*” OR “Pea Family” OR legumes OR grape OR “*Vitis vinifera*”). The specific search strings used for each database are detailed in Supplementary Table S1.

The initial search yielded 605 articles. After adding 1 article identified through preliminary searches and removing 180 duplicates, 426 unique records remained. Two authors (S.T. and L.Y.) independently screened the titles and abstracts of these 426 records to assess eligibility for inclusion. Full-text articles were retrieved for review if deemed potentially eligible by at least one reviewer, or if insufficient information was available in the title/abstract to make a decision. This process identified 77 articles for full-text retrieval. Full text could not be obtained for 3 articles. The remaining 74 full-text articles were then independently assessed for eligibility by the same two authors (S.T. and L.Y.). Any disagreements regarding inclusion were resolved through discussion between the reviewers; involvement of a third arbitrator was not required.

### 2.2. Optimization of PE Protein Components

We included studies of any design that assessed prime editing efficiency in crops. Specific inclusion and exclusion criteria were established based on the review objectives (see Supplementary Table S2).

**Figure 1:**
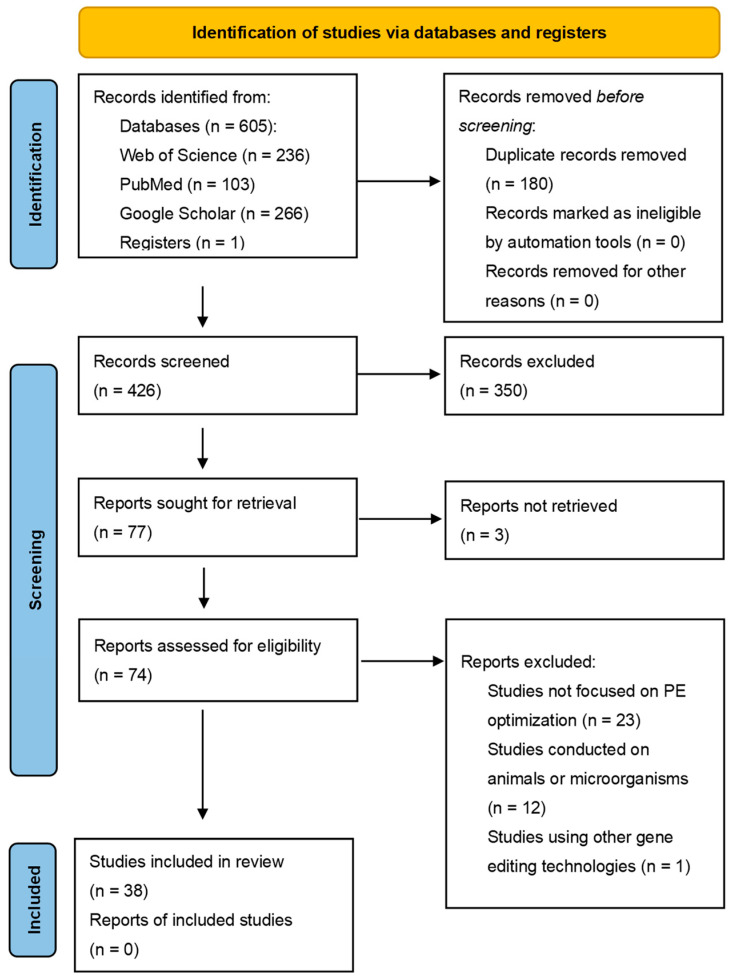
*PRISMA* flow diagram.

Applying these criteria to the literature retrieved from the three databases resulted in the final inclusion of 38 eligible articles. The complete study selection process is illustrated in the *PRISMA* flowchart (see Figure 1).

### 2.3. Data Management and Extraction

Data from all included studies were extracted by L.Y. using a pre-designed and piloted data extraction form. The extracted data were then independently checked for accuracy and completeness by S.T. The extracted information encompassed review characteristics (study title, publication date), study subjects (species), *PE* system used, edit type, editing efficiency, accuracy, efficiency enhancement strategies employed, and effects achieved. Any discrepancies in the extracted data were resolved through comparison and discussion. Where necessary, authors of the identified studies were contacted to obtain missing information.

### 2.4. Review Quality Assessment

The methodological quality of each included study was independently assessed by two reviewers using a quality checklist. Any disagreements in quality assessment were resolved through consensus discussion or, if needed, by consulting a senior member of the review team (see Supplementary Table S3).

## 3. Results

This systematic review ultimately included 38 eligible published articles (see Figure 1 *PRISMA* flowchart for the screening process). Comprehensive analysis of these studies revealed a notable concentration of current plant *PE* research in specific species, with rice being the predominant focus. To systematically present the extensive data extracted from these publications, we constructed a master data table containing all independent experimental data points (detailed in Supplementary Table S4). Our subsequent discussion will be supported by a series of summary tables and figures (Table 1, Table 2, Table 3, Table 4, Table 5, Table 6 and Table 7). Based on this foundation, we delineate the developmental trajectory of *PE* technology in plants, organized into three main levels reflecting the evolution of its core efficacy.

### 3.1. Working Mechanism and Foundational Functional Validation of Prime Editing

**Figure 2:**
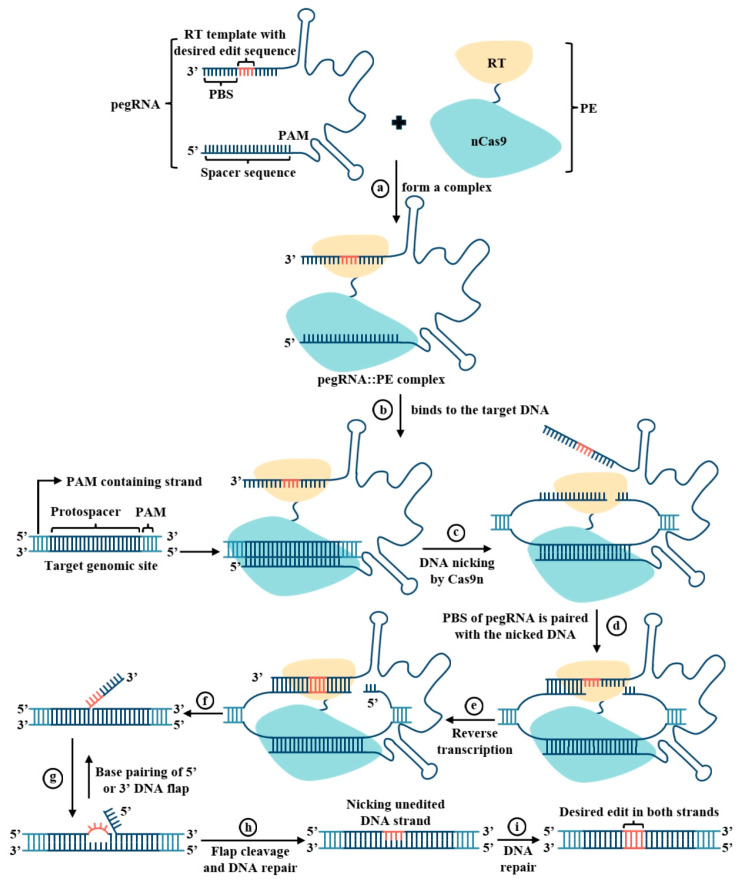
Schematic illustration of the *PE* system components and mechanism. Color/symbol code: *nCas9* (*H840A* variant, light blue) targets the *PAM*-containing strand (blue). Reverse transcriptase (*RT*, yellow) synthesizes the edited strand. *pegRNA*: spacer (marked in figure), *PBS* (marked in figure), and *RTT* (orange). Edited *DNA*: orange mark new edits. Key terms: *PAM* (Protospacer Adjacent Motif) on the non-target strand; *RTT* (Reverse Transcriptase Template) encodes edits with flanking homology; 3′ flap (edited strand) is preferentially retained due to higher homology.

As introduced in the Introduction, *PE* is a revolutionary “search-and-replace” genome editing tool. Its intricate and sophisticated working mechanism—utilizing an *nCas9-RT* fusion protein and a specially engineered *pegRNA* to achieve precise editing without inducing *DSBs*—is detailed in Figure 2. Building upon this innovative mechanism, researchers promptly validated its diverse editing capabilities in plants.

Following the proposal of this innovative mechanism, researchers rapidly applied it to plants to validate its diverse editing capabilities. Early studies primarily focused on the foundational *PE2* system and its plant-optimized versions (e.g., *pPE2*), testing its functionality in various important crops such as rice, wheat, maize, and tomato. To visually demonstrate the fundamental capabilities of *PE* technology, we selected key representative examples from the literature and systematically compiled them in Table 1. Concurrently, a comprehensive dataset detailing editing efficiencies across different species, targets, and systems from all included studies is organized in Supplementary Table S5 for full reference.

As evidenced by the data in Table 1, even the early-stage *PE2* platform exhibited robust and versatile precise editing capabilities. For instance, the *pPE2* system successfully introduced various single-base substitutions in rice protoplasts, with efficiencies reaching up to 5.7%; it also mediated the insertion of a three-bp fragment (2% efficiency) and the deletion of a six-bp fragment (8.2% efficiency) [9]. Similarly, the same system achieved single-base substitutions in wheat protoplasts with efficiencies up to 1.4% [9]. In regenerated transgenic rice plants, the *pPE2* system not only enabled multiple base substitutions but also mediated the insertion of a three-bp small fragment with an efficiency as high as 19.8% [14]. Particularly noteworthy, Li et al. (2022) achieved a remarkably high base substitution efficiency of 66.7% at the rice *Waxy* locus and completed a six-bp fragment insertion with 36.8% efficiency using a “simplified” *PE2* system with meticulously designed *pegRNAs* [13]. Exploring different gene loci, Zong et al. (2022) accomplished a deletion as long as 18 bp with 2.80% efficiency, further confirming the editing potential of the *PE2* system [19].

Although these early achievements established the foundation for *PE* technology as a versatile editing tool in plants, the data in Table 1 simultaneously highlight significant variability in editing efficiency across different species, target sites, and edit types. For example, *PE2* efficiency is typically very low in legume crops and tomato [15,16], contrasting sharply with its high performance in rice. Even within rice for single-base substitutions, editing efficiency at the *OsCDC48* target gene reached 29.17%, whereas no effective editing was detected for the *OsACC1* target gene [17]. For editing the identical site in the *OsPDS* gene, A-to-T substitution efficiency reached 31.3%, but A-to-C substitution failed to produce the intended edit. Furthermore, for the same single-base substitution at the *OsACC1* gene, efficiency varied widely from 0.0% to 14.6% depending on which of four different *pegRNAs* was used [14]. These results underscore the core challenge of unstable efficiency inherent in the *PE* system, which became a primary focus for subsequent optimization efforts.

### 3.2. Core Challenge of PE: Overcoming the Bottleneck of Low Efficiency

Despite its functional versatility (as described in Section 3.1), *PE* in plants is consistently hampered by issues of low and variable efficiency, constituting the primary bottleneck impeding its widespread application. To address this, researchers have undertaken systematic optimization from multiple angles. These optimization strategies can be broadly categorized into four main classes: (1) Engineering the core editing components; (2) Regulating the expression and delivery of the entire *PE* system; (3) Optimizing the editing reaction process; and (4) Efficient screening and enrichment of edited products. This section elaborates on strategies across these four levels.

#### 3.2.1. Engineering Core Editing Components

The most direct approach to enhancing *PE* system performance involves engineering its core molecular “parts”—namely, the editor protein and the *pegRNA*—to augment their intrinsic catalytic activity, stability, and specificity.

**Optimization of the Editor Protein.** Engineering the core protein component of the *PE* editor is a primary strategy to boost its catalytic activity. Analysis of the included literature reveals that modification approaches primarily include optimizing the *Cas9* moiety, engineering the *RT* component, and introducing new elements or reconfiguring the overall architecture. The specific performance of these strategies across different species and editing tasks is summarized in Table 2.

First, optimizing the *Cas9* moiety is a direct means to improve *PE* system performance. For instance, the PEmax architecture, incorporating mutations like *R221K/N394K* into *SpCas9* (*H840A*) and optimizing the nuclear localization signal (*NLS*), aims to enhance editor binding to *pegRNA* and nuclear import efficiency. Testing in rice demonstrated that this strategy effectively increased editing frequency by 3.80- to 5.35-fold [17]. However, directly substituting the Cas protein presents challenges. For example, Hua et al. (2020) attempted to replace *SpCas9* (*H840A*) with *SaCas9* (*N580A*), which resulted in almost no detectable editing events. This finding suggests that systematic co-optimization is required to harness the editing capability of different *Cas* proteins [20].

Second, engineering the *RT* component represents the most fruitful and diverse area of current progress, primarily manifested in two aspects:

(1) Adjusting its spatial conformation and structure. Xu et al. (2022) found that relocating the *RT* enzyme from the traditional C-terminal fusion to an N-terminal fusion position favored the reverse transcription process in plant cells. This modification increased editing efficiency from 0–3.5% to 2.6–14.3% across multiple rice sites [21]. Zong et al. (2022) developed the *ePPE* system, which enhanced complex stability and editing capability by deleting the RNase H domain of *RT* and fusing it with a viral nucleocapsid (*NC*) protein. This resulted in average efficiency improvements of 3.9-fold for base substitutions and 6.5-fold for deletions, and enabled long fragment insertions unattainable with traditional *PPE* [19];
(2) Optimizing or replacing the *RT* enzyme. Introducing point mutations is an effective “fine-tuning” strategy. For example, Ni et al. (2023) introduced the *V223A* mutation into *M-MLV RT*, which boosted the efficiency of various editing tasks by an additional 1.2- to 5.3-fold [22]. Replacing the *RT* source is more complex. While attempts by Lin et al. (2020) using *CaMV RT* or retron-derived *RT* resulted in reduced efficiency [9], Cao et al. (2024) found that an optimized *Tf1 RT* could increase average efficiency by 3.5-fold [23]. Paradoxically, Xu et al. (2024) reported that using *Tf1 RT* decreased efficiency [24]. These seemingly contradictory results underscore the complexity of *PE* optimization, where outcomes are highly dependent on the specific system, target site, and edit type. Furthermore, Cao et al. (2024) demonstrated that employing dual *RT* modules could synergistically further enhance editing efficiency [23].

Additionally, researchers have developed several atypical molecular engineering strategies. One approach involves introducing auxiliary proteins. For instance, Liang et al. (2023) co-expressed *T5* exonuclease alongside the *PE2* components within the vector, significantly boosting editing efficiency by facilitating the *DNA* repair process. This led to a five-fold increase in the proportion of homozygous mutants in rice [25]. Another approach focuses on reconfiguring the entire editor architecture. Addressing the limitation of excessively large traditional fusion proteins, Lu et al. (2025) developed a modular PE (*mPE*) system. This system splits the fusion protein into three independently expressed components, dramatically enhancing editing efficiency. In tobacco, *mPE* increased overall *PE* efficiency by 26.4-fold, with specific edit types showing efficiency gains as high as 1288-fold [26].

**Design and Stability Optimization of *****pegRNA*****.** As the “navigation map” of the *PE* system, the sequence design and stability of *pegRNA* directly determine editing efficiency. Optimization strategies primarily encompass two levels, with specific details summarized in Table 3.

First, at the level of rational element design, Lin et al. (2021) discovered a strong correlation between *PBS* Tm value and editing efficiency. Multiple experiments indicated that a *PBS* Tm of 30 °C yielded the highest *PE* editing efficiency in rice, following a normal distribution [27]. Next, they developed the *dual-pegRNA* strategy, employing two independent *pegRNAs* encoding the same edit in forward and reverse orientations. This significantly increased editing efficiency, achieving up to 24.5% efficiency at tested sites, with average efficiency being 4.2-fold higher (up to 27.9-fold) than using the forward *pegRNA* alone and 1.8-fold higher (up to 7.2-fold) than the reverse *pegRNA* alone [27]. Addressing the issue of increased byproducts associated with efficient editors, Jiang et al. (2022) proposed a reverse transcriptase template (*RTT*) termination design principle. This principle requires the *RTT* to terminate 1–3 bp downstream of specific genomic bases (*C/GC/TGC*), a strategy that successfully eliminated byproducts [28]. Notably, *pegRNA* secondary structure has been proven to be a critical factor influencing efficiency. Li et al. (2022) found that minor differences in the *PE* template sequence, leading to disruption of key functional stem-loop structures in the *pegRNA*, could cause editing efficiency to plummet from 66.7% to 0%. This highlights that avoiding template hairpins/spacer complementarity and maintaining the integrity of the *gRNA* conserved domains are paramount prerequisites during *pegRNA* design [13]. Furthermore, Xu et al. (2022) developed the *RT-M* strategy, which introduces both the desired mutation and a nearby synonymous mutation synchronously. This approach achieved efficiency leaps at targets like *OsALS-1* (0%→4.3%) and *OsACC-2* (0.5%→4.4%) [21]. Lou et al. (2025) proposed target site selection principles for editing: avoid functional cis-acting elements, prioritize open chromatin regions, and choose sites near the translation start site. Applying these principles successfully optimized traits in various tomato and rice quality genes [29].

Second, at the level of enhancing *pegRNA* stability, the addition of structured *RNA* motifs (forming *epegRNA*) has become a gold standard. This strategy significantly increases the effective intracellular concentration of *pegRNA* by protecting its 3′ end from degradation. Li et al. (2022) reported that adding the evopreQ1 *RNA* motif resulted in a 2.35- to 29.22-fold increase in mutation frequency across all four tested sites [17]. Ni et al. (2023) also enhanced *pegRNA* stability using the *Csy4* nuclease system. *Csy4* nuclease binds its recognition site and cleaves the fused transcript to release the *pegRNA* and single guide *RNA* (*sgRNA*). The *Csy4* recognition site, retained at the 3′ end of the *pegRNA* after cleavage, forms a hairpin structure that protects its stability. This system achieved an average efficiency of 13.8% and enabled multiplex editing of 4–10 genes with average efficiencies ranging from 7.4% to 10.3% [22].

#### 3.2.2. Expression Regulation and Efficient Delivery of PE Systems

Once high-performance “parts” are available, the next step is ensuring they can be produced abundantly and efficiently within the cell. This section focuses on strategies aimed at increasing the intracellular concentration of all *PE* system components, primarily covering the optimization of expression elements (e.g., promoters) and innovations in expression vectors and delivery systems. Key information on the strategies discussed in this section for enhancing *PE* expression and delivery efficiency is summarized in Table 4.

**Optimization of Expression Elements: Enhancing Transcriptional Efficiency.** In optimizing expression elements to enhance transcriptional efficiency, researchers have pursued two parallel directions: one optimizing *PE* protein expression, often coupled with selection strategies, and the other specifically boosting the expression abundance of the critical *pegRNA*.

First, regarding optimizing *PE* protein expression, Xu et al. (2020) significantly increased editing efficiency in rice from 0–1.2% to 2.6–26% by employing the maize ubiquitin gene promoter *Zmubi1* in conjunction with hygromycin selection [10]. Similarly, Lu et al. (2021) used the *AtRPS5A* promoter in tomato, elevating average editing efficiency from 0.85% to 2.6% [12]. These results collectively indicate that identifying and applying highly active promoters is a key factor in enhancing *PE* protein expression and consequently improving editing efficiency across diverse plant species.

Second, for optimizing *pegRNA* expression, researchers have explored more diverse strategies due to its unique structural requirements. Simply increasing the copy number of the *pegRNA* expression cassette (doubling) yielded inconsistent results. Jiang et al. (2020) observed no significant efficiency improvement with the doubling strategy in their system [11], while Qiao et al. (2023) achieved an increase in homozygous editing efficiency from 0% to 1.3% in maize [30]. This discrepancy suggests that merely increasing expression cassette copies may not be a universal solution, as its effectiveness appears dependent on the species or specific experimental setup. More effective strategies focus on optimizing the promoter itself. Jiang et al. (2020) pioneered the use of a *U6* composite promoter driven by the *CaMV 35S* promoter and *CmYLCV* enhancer (*35S-CmYLCV-U6*) in maize, successfully increasing editing efficiency from 0.8–4.9% to 1.9–7.1% [11]. The efficacy of this approach was subsequently validated by Li et al. (2022) in rice, where the U6 composite promoter enhanced editing efficiency by 1.66- to 15.60-fold [17]. Furthermore, Biswas et al. (2022) demonstrated that the *CmYLCV* promoter is crucial for achieving successful editing in legume crops [15]. To overcome the limitations of *Pol III* promoters in dicot plants, Lu et al. developed a *Pol II* transcription system based on a *tRNA* processing system and the *AtUb10* promoter. This system not only enabled successful editing but also circumvented premature termination caused by internal poly-T sequences in the template, boosting *pegRNA* expression levels by more than 20-fold and providing a richer substrate pool for the *PE* reaction [26,31].

**Innovation in Expression Vectors: Amplifying Editing Tools.** Beyond promoter optimization, researchers have also innovated *PE* expression vectors. Wang et al. (2021) attempted to co-deliver the *PE* vector with a separate vector expressing *pegRNA*/*sgRNA* to increase *pegRNA* concentration in the reaction system, but observed no significant change in editing efficiency [32]. Vu et al. (2024) constructed *PE* vectors using the bean yellow dwarf virus (*BeYDV*) replicon system. This system autonomously amplifies its cargo *DNA*, resulting in a 1.3-fold average increase in expression cassette *DNA*, a 1.9–2.0-fold increase in *RNA* transcripts, and a 4.5-fold increase in *PE* protein levels. Consequently, the desired *PE* efficiency was enhanced by 6.6–7.8-fold compared to standard *T-DNA* delivery [33].

#### 3.2.3. Optimization and Regulation of the Editing Reaction Process

Once the *PE* tools are in place within the cell, creating an optimal “working environment” for them is equally crucial. The strategies discussed in this section do not alter the *PE* tools themselves but, instead, modulate endogenous cellular pathways or external physical conditions to support the smooth progression of the editing reaction. Relevant information discussed in this section is summarized in Table 5.

**Modulation of the Intracellular Endogenous Environment.** Modifying the intracellular endogenous environment offers an effective efficiency-boosting pathway independent of the *PE* tools. Research indicates that these strategies primarily focus on two aspects: regulating *DNA* repair pathways to reduce byproducts and preserve desired edits, and modulating chromatin structure to enhance the accessibility of the target site.

Research on regulating the *DNA* mismatch repair (*MMR*) pathway to improve plant gene editing efficiency has yielded a key and intriguing finding: strategies effective in mammalian cells often underperform in plant systems. For instance, the *PE3* system, designed to guide repair by introducing a second nick, not only failed to consistently increase editing efficiency in plants but also significantly increased the frequency of undesired *NHEJ*-mediated mutations [9,13,14,34]. This phenomenon suggests fundamental differences in *DNA* damage repair mechanisms between plant and animal cells. Although the subsequently developed *PE3b* system partially alleviated the *NHEJ* mutation issue, its application still faced limitations such as low editing efficiency [14] or the generation of other unintended byproducts [34]. Notably, Jiang et al. (2022) confirmed through extensive *sgRNA* screening that *PE3* efficiency gains are highly dependent on selecting specific and highly efficient *sgRNA*s, highlighting the complexity of optimizing *PE3/3b* systems [28]. Another research avenue involves suppressing key *MMR* proteins by co-expressing dominant-negative mutants (e.g., *hMLH1dn* or *OsMLH1dn*). However, multiple studies [17,28,35] found that this strategy failed to effectively enhance *PE* efficiency in various plant systems and could even inhibit editing at certain targets. Paradoxically, Qiao et al. (2023) reported that fused expression of *ZmMLH1dn* significantly boosted editing efficiency in maize (from 2.2% to 12%) [30]. These divergent outcomes collectively suggest that *MMR* suppression strategies based on *MLH1dn* exhibit significant instability and species/target site dependency in plants. Addressing these challenges, Liu et al. (2024) achieved a significant breakthrough with their transient suppression system based on *OsMLH1*-specific ihp*RNA* (intron-containing hairpin *RNA*). By temporarily downregulating *MMR* pathway activity, this method successfully increased average editing efficiency by 1.51-fold and raised the proportion of plants obtaining edits from 71.53% to 87.15%. Crucially, the researchers integrated a *Cre-LoxP* recombination system to enable effective excision of the *MMR* interference module, thereby mitigating potential risks associated with long-term *MMR* suppression, such as reduced fertility [35].

Additionally, enhancing target chromatin accessibility represents another important endogenous regulatory pathway. Opening up tightly packed chromatin structures can facilitate easier access of the *PE* protein to the *DNA* target, thereby increasing editing efficiency. For example, Bai et al. (2024) introduced an *hFTO* box into the enp*PE2* system. Overexpression of *hFTO* promotes chromatin opening and alleviates gene expression suppression, significantly increasing average editing efficiency from 33.49% to 52.48% and nearly doubling homozygous mutation efficiency (13.71%→26.88%). However, this strategy came at the cost of a mild increase in off-target editing frequency [36].

**Optimization of External Physical Conditions.** Among external condition modulations, temperature has proven to be a simple yet effective optimization lever. Lin et al. (2020) found editing efficiency in rice protoplasts was significantly higher at 37 °C (6.3%) than at 26 °C (3.9%) [9]; Zou et al. (2022) achieved a 3.1–3.7-fold efficiency increase by applying a short-term 42 °C heat shock for 2 h to rice callus [37]; Vu et al. (2024) increased the culture temperature for tomato from 25 °C to 34 °C, boosting efficiency by 2.9–3.2-fold [33]; and Lu et al. (2024) developed the *RHTT* cycling strategy (2 h at 37 °C + 6 h at 25 °C cycle), which enhanced editing efficiency in tobacco by up to 16.3-fold [31].

#### 3.2.4. Enrichment and Efficient Screening of Edited Events

Once the editing reaction is complete, rapidly and accurately identifying rare positive events from a vast population of cells is a critical step in determining the technology’s applicability. This section introduces strategies for enriching and identifying edited cells by incorporating selection markers. Key information is summarized in Table 6.

**Innovative Screening Systems Provide Crucial Support for Enriching Edited Events.** In antibiotic selection systems, hygromycin selection boosted rice editing efficiency from 0–1.2% to 2.6–26% [10]; other studies corroborated the effectiveness of this strategy, achieving efficiency increases from 0% to 16.7% [14]; a dual-selection system (Bispyribac + hygromycin) increased rice *PE3* efficiency from 0–1% to 3.2–54.2% [38]. For visual screening, Zhang et al. (2023) developed an anthocyanin accumulation-based screening system. This increased editing efficiency at the tobacco *ALS-like-T* locus from 6.5% to 13.0% and at the *NIP2–1-T* locus from 7.5% to 16.3%, while also making the screening process more intuitive and efficient [39].

### 3.3. Functional Expansion and Advanced Applications of PE

With the significant improvement in the core editing efficiency of *PE* systems (as described in Section 3.2), researchers have begun to transcend the limitations of simple base substitutions or small *indels*, striving to expand the capability boundaries of *PE* technology to meet the demands of complex genome editing and challenging breeding scenarios. Current frontier explorations focus on two major directions: (1) expanding the editing scope and enhancing practical applicability; and (2) developing advanced system architectures capable of achieving large-scale, multifunctional, and complex edits. These advanced strategies and their applications are summarized in Table 7.

#### 3.3.1. Expanding Editing Scope and Enhancing Applicability

The enhanced practicality of *PE* technology is first reflected in the expansion of its targeting scope. To overcome the reliance of traditional *SpCas9* on *NGG PAM* sequences, researchers have utilized *SpCas9* variants with broader *PAM* recognition specificities, such as *SpG, SpRY*, and *ScCas9*. As shown in Table 7, Lin et al. (2021) and Zong et al. (2022) achieved editing at *NG PAM* sites using *SpG*, with efficiencies of 1.9% [27] and 0.4%–7.5% [19], respectively. Sun et al. (2024), combining *SpG* and *SpRY* variants with the *dual-pegRNA* strategy, observed significant *PAM* dependency in editing efficiency: *NGC + NGC* combinations yielded the highest efficiency, followed by *NGC + NGT*, *NGT + NGT*, and *NGC + NGA* combinations [40]. Li et al. (2025) employed *SpRY* and *ScCas9* to expand editable sites. While *SpRY* offers high *PAM* flexibility (recognizing *NRN* or *NYN* targets), it exhibits high self-editing rates (33%–64%), resulting in low editing efficiencies of only 2.38%–6.25%. In contrast, *ScCas9* recognizes *NNG PAM* sites without self-editing issues, enabling targeting of nearly 100% of rice genes and achieving editing efficiencies of 20%–70.83% [41].

Second, efforts to enhance application convenience focus on simplifying breeding workflows and obtaining transgene-free plants. Zou et al. (2025) constructed a *Cas9-PE* system by replacing *nCas9* with active *Cas9*. This system concurrently achieves precise editing and site-specific random mutations, successfully generating transgene-free T0 rice plants, albeit at the cost of reduced precise editing frequency [42]. Lu et al. (2025) integrated three core technologies: *PE* structural optimization (incorporating the *Csy4* system and *RT* variants), enhanced Agrobacterium (carrying an additional Vir gene cluster), and a pyroxsulam selection system. This approach achieved, for the first time, Agrobacterium-mediated transient *PE*, generating transgene-free co-edited T0 rice plants in a single step. These methods provide efficient tools for molecular design breeding [43].

#### 3.3.2. Achieving Complex and Multifunctional Editing

Expanding *PE* technology’s capability to handle large-scale, complex genome editing is a critical direction for the field, with significant breakthroughs achieved in long fragment manipulation. Enhancements in basic capability are exemplified by the *ePPE* system, an improvement over *PPE*, which enabled insertions of specific lengths (18 bp, 24 bp, 34 bp) with efficiencies of 0.2–3.1% [19], and the *NM-PE* system, which efficiently inserted a 44-bp tag using wild-type *Cas9* (55.00–56.25% efficiency) [41]. Addressing more complex replacement needs, the *GRAND* editing strategy significantly improved the precision of long fragment replacement by designing two partially overlapping, non-homologous reverse transcription templates. This strategy successfully replaced 57 bp, 90 bp, or 186 bp sequences with a 72 bp target sequence at efficiencies of 8.33–25% [44]. Xu et al. (2024) constructed the *PE6d* system by integrating the *GRAND* strategy, further enhancing tag insertion capability to efficiently insert tags ranging from 27 bp to 90 bp, supporting insertions of up to 135 bp [24]. Larger fragment insertions were realized by the *TJ-PE* system, which achieved insertions of fragments up to 1002 bp, with an efficiency of 12.6% [45]. For long fragment deletions, the *PRIME-Del* strategy performed notably well, enabling precise deletion of fragments ranging from 50 bp to 2000 bp with efficiencies of 37.5–84.2% (heterozygous) and 14.3–63% (homozygous) [46]. To achieve precise manipulation of very large fragments (kb-Mb scale), researchers developed systems engineering strategies. The PrimeRoot system innovatively integrated Cre-Lox66/Lox71 recombinase with an *ePE-dual-epegRNA* vector. This system accomplished 1.4 kb and 4.9 kb insertions in regenerated rice plants and achieved insertions of up to 11.1 kb in protoplasts [40]. The *DualPE* system represents the current pinnacle of capability, demonstrating powerful cross-species editing. It can generate specific deletions of ~500 bp to 2 Mb in wheat, directly replace a 258 kb fragment, invert a 205.4 kb fragment, and achieve large fragment editing efficiencies as high as 72.7% in tobacco and tomato [47].

## 4. Discussion

### 4.1. Interpretation of Key Findings: From Research Strategy to Functional Evolution

This systematic review delineates the developmental trajectory of *PE* technology in plants since its inception, revealing progress across three interconnected core dimensions.

The first dimension encompasses the foundational validation of technical feasibility. Early systems, exemplified by *PE2*, demonstrated the theoretical viability of diverse edits in plants. They successfully achieved all types of single-base substitutions and small-fragment edits in model crops like rice, with observed efficiencies reaching up to 66.7% for single-point substitutions. However, these pioneering efforts also exposed the technology’s initial limitations: its efficiency exhibited strong dependence on species, target site, and edit type, resulting in significant unpredictability.

The second critical dimension involves systematic optimization aimed at enhancing efficiency and reducing costs. To address the bottlenecks of low and unstable efficiency, researchers have driven improvements on four key fronts. These include engineering core components like editor proteins and *pegRNA*, strengthening expression systems, modulating the cellular reaction environment, and enabling the efficient enrichment of positive editing events. These concerted efforts have systematically enhanced the overall performance of *PE* technology.

The third frontier dimension focuses on functional expansion and advanced applications, building upon improved efficiency. By integrating *Cas9* variants with expanded *PAM* compatibility (e.g., *SpG, SpRY, ScCas9*), the targetable scope of *PE* has been dramatically broadened. More significantly, the development of sophisticated system architectures (e.g., *GRAND* editing, *PRIME-Del*, PrimeRoot, and *DualPE*) has enabled complex genome edits that were previously unattainable. These include precise long-fragment replacements, large-fragment in situ insertions, and even megabase (Mb)-scale chromosomal manipulations.

In summary, the evolution of *PE* technology in plants follows a path from “theoretically feasible” to “efficiently usable,” and ultimately to “functionally powerful.” It has transformed from a basic editing tool into a robust platform that supports complex genome design. This evolution provides unprecedented technological support for precision crop breeding.

### 4.2. Limitations of the Included Studies and Current Research Challenges

While this review maps rapid advancements, it is also essential to critically discuss limitations. The included studies and the broader research field both have inherent challenges.

Firstly, the primary literature shows significant variability across species. This research is overwhelmingly focused on rice. Consequently, many promising optimization strategies have not been validated in other staple crops like wheat and maize. Furthermore, application data for dicotyledonous cash crops are particularly scarce. This species imbalance means *PE* efficiency remains limited and unproven in many important crops, restricting the general applicability of the findings.

Secondly, the included studies show a pronounced “efficiency-first” bias. Most research focuses on short-term gains in laboratory or greenhouse settings, creating a critical lack of field-level validation. This results in a significant knowledge gap in several key practical areas. These include the long-term genetic stability of edits, their real-world impact on agronomic traits, and their overall genome-wide safety profiles. Addressing these gaps through rigorous field trials is imperative. Such trials are a prerequisite for both biosafety assessment and any widespread breeding applications.

### 4.3. Future Perspectives and Outstanding Questions

Looking forward, the development of plant *PE* technology will concentrate on two primary directions: broadening the application scope and continuously optimizing core performance. The ultimate goal is to achieve more efficient, precise, and predictable crop genetic improvement.

In terms of application expansion, *PE* is transitioning from the laboratory into scenarios with tangible breeding value, although the depth of application varies markedly between species. In rice, its application is relatively mature, extensively covering crucial breeding objectives such as herbicide resistance, pest/disease resistance, plant architecture improvement, enhanced stress resilience, and nutritional quality fortification [29,48,49,50]. Notably, *PE* can now address specific industry challenges through sophisticated molecular design. A prime example is research on tobacco secondary metabolites. For example, a precise G-to-T conversion in the tobacco NtCPS2 gene successfully restored its function. This enabled the de novo biosynthesis of the high-value compound Z-abienol. This trait was stably inherited, significantly enhancing the crop’s economic value [39]. Similarly, editing the *Sl*Lin5 gene in tomato improved fruit flavor [51], while editing the *VvDXS1* gene in grape imparted a novel muscat flavor [52]. These cases demonstrate that *PE* technology has entered an era of precision breeding capable of creating tangible economic value. Future research should prioritize adapting these successful strategies to a wider range of crop systems.

Specifically, expanding *PE* applications to other major crop categories holds immense promise. In legumes, such as soybean, *PE* could be used to precisely edit fatty acid desaturase genes (e.g., *FAD2*) to create high-oleic oil with improved stability, or to knock out genes encoding anti-nutritional factors like phytic acid. For horticultural crops, the potential is equally vast. In potato, one could target polyphenol oxidase (*PPO*) genes to prevent enzymatic browning. In fruits like strawberry or tomato, editing genes within the ethylene biosynthesis pathway or key ripening transcription factors could significantly extend shelf life and improve transportability. Furthermore, the precise nature of *PE* makes it an ideal tool for fine-tuning metabolic pathways to enhance flavor, fragrance, or the content of valuable phytonutrients in a wide array of fruits and vegetables.

In terms of technical optimization, enhancing efficiency and precision will require adopting advanced strategies, many of which have already shown breakthroughs in human cell studies. Key frontier directions include the following:

(1) Fusing endogenous small RNA-binding proteins (e.g., La) to stabilize *pegRNA* and boost editing activity [53];
(2) Using AI-driven rational design to optimize reverse transcription templates (*RTTs*). This can also be used to develop *PE* systems with a reverse editing window, which would expand the editable region and improve precision [54];
(3) Developing innovative delivery platforms based on pseudoviral particles to enable more efficient and safer delivery of editing tools [55];
(4) Constructing inverse *PE* platforms based on circular *RNA* to circumvent limitations inherent in traditional editing orientations [56].

Introducing these innovative concepts into plant systems promises to provide novel solutions for overcoming current technical bottlenecks. Ultimately, this will propel *PE* technology toward becoming a standardized and versatile core tool for precision crop breeding.

## Figures and Tables

**Table 1:** Diverse Editing Examples of the *PE2* Baseline System in Plants.

Species	Target Gene	Edit Type	*PE* System	Experimental System	Editing Efficiency	Remarks	Reference
Rice	*OsCDC48*	Deletion (6 bp)	*PPE2*	Protoplast	8.20%	Achieved 6-bp deletion in rice protoplasts	[9]
Rice	*OsCDC48*	Insertion (3 bp)	*PPE2*	Protoplast	2.00%	Achieved 3-bp insertion in rice protoplasts	[9]
Rice	*OsCDC48*	Base substitution (1 bp)	*PPE2*	Protoplast	5.70%	Highest efficiency among multiple single-base substitutions	[9]
Wheat	*TaGASR7*	Base substitution (1 bp)	*PPE2*	Protoplast	1.40%	Highest efficiency among multiple single-base substitutions in wheat protoplasts	[9]
Rice	*OsPDS*	Insertion (3 bp)	*pPE2*	Callus	19.80%	Achieved small-fragment insertion in rice plants	[14]
Rice	*OsWx*	Base substitution (3 bp)	*PE2*	Callus	66.70%	High-efficiency base substitution in rice plants	[13]
Rice	*OsWx*	Insertion (6 bp)	*PE2*	Regenerated plants	36.80%	Achieved 6-bp insertion with relatively high efficiency	[13]
Rice	*OsRDD1*	Deletion (18 bp)	*PPE*	Regenerated plants	2.80%	Successfully achieved 18-bp deletion, albeit at low efficiency	[19]
Legume crops	*Exogenous mutant GFP*	Base substitution (1 bp)	*PE2*	Protoplast	0.00%	*PE2* system failed to edit legume crop protoplasts	[15]
Tomato	*SlWH9*	Base substitution (1 bp)	*nCas9-RT*	Callus	0.11%	Highest editing efficiency in tomato callus, yet still suboptimal	[16]
Rice	*OsCDC48*	Base substitution (1 bp)	*pPE2*	Regenerated plants	29.17%	Significant variation in *PE2* efficiency across different target genes	[17]
Rice	*OsACC*	Base substitution (1 bp)	*pPE2*	Regenerated plants	0.00%	—	[17]
Rice	*OsPDS*	Base substitution (A to T)	*pPE2*	Regenerated plants	31.30%	Markedly different efficiencies for different edits at the same locus	[14]
Rice	*OsPDS*	Base substitution (A to C)	*pPE2*	Regenerated plants	0.00%	—	[14]
Rice	*OsACC*	Base substitution (G to C)	*pPE2*	Regenerated plants	14.60%	Efficiency variation using different *pegRNAs* for the same edit at the same locus	[14]
Rice	*OsACC*	Base substitution (G to C)	*pPE2*	Regenerated plants	3.10%	—	[14]
Rice	*OsACC*	Base substitution (G to C)	*pPE2*	Regenerated plants	1.00%	—	[14]
Rice	*OsACC*	Base substitution (G to C)	*pPE2*	Regenerated plants	0.00%	—	[14]

**Table 2:** Summary of Engineering Strategies to Enhance Prime Editor Protein Performance.

Component	Specific Strategy	Baseline	Edit Type	Key Effect	Species	Reference
*nCas9*	*PE*max architecture	*pPE2*	Base sub (1-bp or 2-bp)	· Introduced *R221K/N394K* mutations enhancing *pegRNA* binding · Efficiency increased 3.80- to 5.35-fold	Rice	[17]
*nCas9*	*SaCas9 (N580A)*	*PE3*	Base sub (1-bp or 2-bp)	· Extremely low editing efficiency, virtually no effective edits	Rice	[20]
*RT*	N-terminal fusion	*PE3*	Base sub (1-bp)	· Efficiency increased: *OsGS3* (3.5%→14.3%), *OsALS-1* (0%→2.6%), *OsACC2* (0%→4.4%)	Rice	[21]
*RT*	*RNase H* domain deletion + *NC* fusion	*PPE*	Base sub (1/2-bp), Del (15–90 bp), Ins (18/24/34 bp)	· Avg base sub efficiency ×3.9 (max ×121.5) · Avg del efficiency ×6.5 · Ins efficiency: 18 bp (3.1%), 24 bp (0.2%), 34 bp (0.3%)	Rice	[19]
*RT*	*V223A* mutation	*ePPE*	Base sub (1-bp), Del (1/5/6-bp)	· Editing efficiency ×1.2–5.3 (avg ×2.8) · No increase in byproducts	Wheat	[22]
*RT*	*CaMV RT* or retron-derived *RT*	*PPE3b*	Base sub (2-bp)	· Efficiency lower than *M-MLV RT*	Rice	[9]
*RT*	*Ec48 RT (PE6a)*, *Tf1 RT (PE6b)*, *Opt Tf1 RT (PE6c)*, *Opt M-MLV RT (PE6d)*	*PE3*	Base sub (1/2/3-bp), Del (84-bp), Ins (30-bp)	· *All PE6* editors except *PE6a* increased efficiency · *PE6c* highest: avg editing ×3.5, homozygous rate significantly increased	Rice	[23]
*RT*	*Tf1 RT*	*ePE2*	Base sub (1-bp), Ins (1/4/27-bp)	· Reduced efficiency for all edit types	Rice	[24]
*RT*	*Dual RT* module (*Tf1 RT*, *Opt M-MLV RT*)	*PE3*	Base sub (1/2/3-bp), Del (84-bp), Ins (30-bp)	· Synergistic effect, efficiency higher than single modules	Rice	[23]
Auxiliary Module	*T5* exonuclease upstream of *Cas9*	*PE2*	Base sub (1/2-bp), Del (4-bp), Ins (4-bp)	· Protoplast efficiency ×1.7–2.9 · Transgenic plant efficiency ×1.34, homozygous mutant ratio ×5	Rice	[25]
Expression System	Modular split (*mPE* system)	*PE2*	Base sub (1/2/4-bp), Ins (3/4/6/7-bp)	· Tobacco transient: Total efficiency 0.01%→0.26% (×26.4); Multi-base ins avg ×197.9 (max ×1288)	Tobacco	[26]

**Table 3:** Summary of *pegRNA* Design and Stability Optimization Strategies.

Category	Specific Strategy	Baseline	Edit Type	Key Effect	Species	Reference
Key Element Design	*PBS Tm* = 30 °C	*PPE2*	Base sub (1/2-bp), Del (2/3-bp), Ins (1-bp)	· *PBS* Tm 30 °C optimal for rice · Efficiency follows normal distribution	Rice	[27]
Key Element Design	*Dual-pegRNA*	*PPE2*	Base sub (1/2-bp), Del (1/2-bp), Ins (1-bp)	· Editing efficiency ×1.8–4.2 · No increase in byproduct ratio	Rice	[27]
Key Element Design	*RTT* termination principle	Unoptimized *pegRNA*	Base sub (1-bp)	· Terminate *RTT* 1–3 bp after *C/GC/TGC* · Completely eliminated byproducts	Rice	[28]
Key Element Design	Optimized secondary structure	*PE2*	Base sub (3-bp)	· Avoid template hairpin/spacer complementarity + maintain *gRNA* conserved domains · Improper structure reduced efficiency from 66.7%→0%	Rice	[13]
Key Element Design	*RT-M* strategy	*PE-P2–RT-S*	Base sub (3/4-bp)	· Introduce primary mutation + adjacent synonymous mutation · Efficiency increased: *OsALS*-1 (0→4.3%), *OsACC-2* (0.5%→4.4%), *OsDEP1* (1.1%→2.6%), *OsWaxy-1* (0→2.2%)	Rice	[21]
Key Element Design	Target selection principle	Unoptimized *pegRNA*	Ins (10-bp)	· Avoid functional elements/select open chromatin/near translation start site · Successfully applied to 4 tomato + 2 rice varieties, boosting yield under normal/heat stress	Tomato, Rice	[29]
Enhanced Stability	Add evopreQ1 *RNA* motif	*pPE2*	Base sub (1/2-bp), Ins (1-bp)	· Mutation frequency ×2.35–29.22	Rice	[17]
Enhanced Stability	*Csy4* nuclease system	*tRNA* system	Simultaneous small-fragment edits	· Avg efficiency 13.8% · Synchronous editing of 4–10 genes: 7.4–10.3%	Wheat	[22]

**Table 4:** Strategies to Enhance *PE* System Expression and Delivery Efficiency.

Category	Specific Strategy	Baseline	Edit Type	Key Effect	Species	Reference
Enhanced Expression—*PE* Protein	*Zm*ubi1 promoter	*OsU6a* promoter	Base sub (1/3/4-bp)	· Combined with hygromycin selection: Editing efficiency increased from 0–1.2%→2.6–26%	Rice	[10]
Enhanced Expression—*PE* Protein	*AtRPS5A* promoter	*35S* promoter	Base sub (3-bp), Del (2-bp), Ins (4-bp)	· Avg editing efficiency 0.85%→2.6%	Tomato	[12]
Enhanced Expression—*pegRNA*	*U6* composite promoter or increased *pegRNA* cassette number	*PE3*	Base sub (1/2/3-bp)	· Editing efficiency 0.8–4.9%→1.9–7.1% · Doubling cassettes did not enhance efficiency	Maize	[11]
Enhanced Expression—*pegRNA*	Doubled *epegRNA* cassette number	*ePE5*max	Base sub (1 bp)	· Homozygous editing efficiency 0%→0.6–1.3%	Rice	[30]
Enhanced Expression—*pegRNA*	*U6* composite promoter	*pPE2*max-evopreQ1	Base sub (1/2-bp), Ins (1-bp)	· Efficiency ×1.66–15.60	Rice	[17]
Enhanced Expression—*pegRNA*	*CmYLCV* promoter	*CAMV 35S/OsU6* promoter	Base sub (1-bp)	· Successful editing only with *CmYLCV* promoter	Legume crops	[15]
Enhanced Expression—*pegRNA*	*Pol II* promoter system (*tRNA* processing)	*Pol III* promoter (*At*U6)	Base sub (1/2-bp)	· *tRNA* processing system + *AtUb10* promoter · Enabled editing in dicots (where *Pol III* failed)	Tobacco	[31]
Enhanced Expression—*pegRNA*	*Pol II* promoter system	*Pol III* promoter	Base sub (1/2/4-bp), Ins (3/4/6/7-bp)	· Unaffected by poly-T sequences in template · *pegRNA* expression ×20 · *Cas9* cutting efficiency (indel rate) ×2–3	Tobacco	[26]
Enhanced Delivery	*pPEG* system	*pPPEM*	Base sub (2 bp), Ins (25 bp)	· Co-transformation with an additional vector expressing *pegRNA/sgRNA* · No significant change in editing efficiency	Rice	[32]
Enhanced Delivery	Geminiviral replicon vector	*T-DNA* vector	N/A	· *DNA* cassette ×1.3 · *RNA* transcript ×1.9–2.0 · *PE* protein level ×4.5 · *PE* efficiency ×6.6–7.8	Tomato	[33]

**Table 5:** Strategies for Optimizing the Prime Editing Reaction Process.

Category	Specific Strategy	Baseline	Edit Type	Key Effect	Species	Reference
*DNA* Repair Pathway	*PE3/PE3b* system	*PE2*	Base sub (1-bp), Del (6-bp), Ins (3-bp)	· Efficiency comparable to *PE2*	Rice, Wheat	[9]
*DNA* Repair Pathway	*PE3/PE3b* system	*PE2*	Ins (3-bp)	· Efficiency comparable to *PE2* · *PE3* prone to large deletions · *PE3b* reduced deletions but introduced other byproducts	Rice	[34]
*DNA* Repair Pathway	*PE3/PE3b* system	*PE2*	Base sub (1-bp)	· *OsACC1* locus: *PE2* (14.6%) vs. *PE3* (18.8% + byproducts) vs. *PE3b* (6.3% no byproducts)	Rice	[14]
*DNA* Repair Pathway	*PE3* system	*PE2*	Base sub (3-bp), Del (2/4/18-bp), Ins (1/2/12 bp)	· Efficiency: *PE3* (2.6–13%) < *PE2* (30–66.7%) · *PE3* induced *NHEJ* byproducts (26.3–38.9%)	Rice	[13]
*DNA* Repair Pathway	*PE3* system	*PE2*	Base sub (1/2/3-bp)	· Protoplast: Avg efficiency ×2.2 at most sites · Reduced byproducts	Rice	[28]
*DNA* Repair Pathway	Fusion *hMLH1dn*	*pPE2*max	Base sub (1/2-bp), Ins (1-bp)	· No significant enhancement	Rice	[17]
*DNA* Repair Pathway	Fusion of various *OsMLH1dn*	*ePE3*max	Base sub (3 bp)	· Did not significantly increase editing efficiency	Rice	[28]
*DNA* Repair Pathway	Fusion *hMLH1dn*, *OsMLH1dn*	*ePE3*	Base sub (1-bp), Del (1-bp), Ins (1-bp)	· No enhancement; Efficiency at *NRT1.1-T* locus reduced by 52%	Rice	[35]
*DNA* Repair Pathway	Fusion *ZmMLH1dn*	*ePE3*max	Base sub (3 bp)	· Homozygous editing efficiency 2.2%→12%	Maize	[30]
*DNA* Repair Pathway	*OsMLH1*-specific *ihpRNA* introduction	*ePE3*	Base sub (1-bp), Del (1-bp), Ins (1-bp)	· Efficiency ×1.30–2.11 (avg ×1.51), no increased off-targets · Edited plant ratio 71.53%→87.15%	Rice	[35]
Chromatin Opening	*hFTO* introduction	*enpPE2*	Base sub (1-bp), Del (2-bp), Ins (1-bp)	· Editing efficiency 33.49%→52.48% · Homozygous mutation frequency 13.71%→26.88% · Mild increase in off-target editing frequency	Rice	[36]
Temperature	37 °C	26 °C	Base sub (1-bp), Ins (3-bp)	· Editing efficiency 3.9%→6.3%	Rice	[9]
Temperature	42 °C treatment for 2 h	34 °C	Base sub (1/3-bp)	· Efficiency ×3.1–3.7	Rice	[37]
Temperature	34 °C	25 °C	N/A	· Efficiency ×2.9–3.2	Tomato	[33]
Temperature	*RHTT* cycle	25 °C	Base sub (1/2-bp)	· 37 °C heat shock for 2 h + 25 °C recovery for 6 h, cycle repeated for 96 h · Precise editing efficiency max ×16.3	Tobacco	[31]

**Table 6:** Strategies for Enriching and Screening *PE* Edited Events.

Category	Specific Strategy	Baseline	Edit Type	Key Effect	Species	Reference
Screening System	Hygromycin selection system	*PE-P1*	Base sub (1/3/4-bp)	· Combined with *Zmubi1* promoter: Editing efficiency increased from 0–1.2%→2.6–26%	Rice	[10]
Screening System	Hygromycin selection system	*pPE2*	Base sub (1-bp)	· Efficiency: 0%→16.7%	Rice	[14]
Screening System	Dual selection system	*PE3*	N/A	· Combined bispyribac-sodium + hygromycin selection outperformed single systems · Efficiency: 0–1%→3.2–54.2%	Rice	[38]
Screening System	Anthocyanin screening system	*PE-Nt3*	Base sub (3/4-bp)	· *PAP1* gene enables visual purple phenotype, facilitating efficient screening · Efficiency: 1.1–7.5%→1.3–16.3%	Tobacco	[39]

**Table 7:** Summary of *PE* Functional Expansion and Advanced Application Strategies.

Category	Specific System/Strategy	Key Capability and Effect	Species	Reference
Expand *PAM* Range	*SpG*	· Targets *NG PAM* · Efficiency up to 1.9%	Rice	[27]
Expand *PAM* Range	*SpG*	· Targets *NG PAM* (*NGC/NGA/NGG*) · Efficiency range 0.4–7.5%	Rice	[19]
Expand *PAM* Range	*SpG or SpRY*	· Targets *NG PAM* · dual-*pegRNA* efficiency: *NGC + NGC > NGC + NGT > NGT + NGT > NGC + NGA > NGT + NGA > NGA + NGA*	Rice	[40]
Expand *PAM* Range	*SpRY*	· High *PAM* flexibility (*NRN/NYN*) · High self-editing rate (33–64%) · Tagging efficiency only 2.38–6.25%	Rice	[41]
Expand *PAM* Range	*ScCas9*	· Targets *NNG PAM* · No self-editing issue · Tagging efficiency 20–70.83% · Targets nearly 100% of rice genes	Rice	[41]
Enhance Usability	Active *Cas9*	· Simultaneous precise editing + random mutation, generating transgene-free T0 plants · Reduced precise editing efficiency	Rice	[42]
Enhance Usability	· Optimized *PE* architecture (*Csy4* system, *RT* variants) · Agrobacterium with extra Vir genes · Pyroxsulam selection	· Achieved transient co-editing, generating transgene-free T0 plants	Rice	[43]
Achieve Complex Edits	*ePPE*	· Extended *PPE* capability: Specific insertion lengths: 18 bp (3.1%), 24 bp (0.2%), 34 bp (0.3%)	Rice	[19]
Achieve Complex Edits	*NM-PE*	· 44 bp insertion efficiency 55.00–56.25%	Rice	[41]
Achieve Complex Edits	*PE6d*	· Significantly increased byproducts for point mutations and small edits · Tag insertion (27–135 bp), but knock-in capacity sharply decreases with tag size	Rice	[24]
Achieve Complex Edits	*GRAND editing*	· Replaced 57 bp, 90 bp, or 186 bp sequences with a 72 bp sequence at 8.33%–25% efficiency	Rice	[44]
Achieve Complex Edits	*TJ-PE*	· Inserted up to 1002 bp at 12.6% efficiency · Combining *Csy4* system + re-added *RNase H* further improved efficiency	Rice	[45]
Achieve Complex Edits	*PRIME-Del*	· Enabled 50 bp–2000 bp deletions · Editing efficiency 37.5–84.2% · Homozygous editing efficiency 14.3–63%	Rice	[46]
Achieve Complex Edits	*PrimeRoot*	· Achieved 1.4 kb, 4.9 kb insertions in regenerated plants · Up to 11.1 kb insertion in protoplasts	Rice	[40]
Achieve Complex Edits	*DualPE*	· Generated specific deletions (~500 bp to 2 Mb) in protoplasts and plants; · Direct replacement of fragments up to 258 kb; · Precise inversion of a 205.4 kb fragment in plants	Wheat	[47]
Achieve Complex Edits	*DualPE*	· Large-fragment *DNA* editing efficiency up to 72.7%	Tobacco, Tomato	[47]

## Data Availability

No new data were created for the production of this manuscript. All of the data discussed and presented here are available in the relevant references cited and listed.

## Supplementary Materials

The following supporting information can be downloaded at https://www.mdpi.com/article/10.3390/genes16080965/s1, Table S1: Details of initial literature search; Table S2: Literature inclusion and exclusion criteria; Table S3: Risk of bias assessment for included primary studies [9,10,11,12,13,14,15,16,17,19,20,21,22,23,24,25,26,27,28,29,30,31,32,33,34,35,36,37,38,39,40,41,42,43,44,45,46,47]; Table S4: Master data table of all extracted information from the included studies [9,10,11,12,13,14,15,16,17,19,20,21,22,23,24,25,26,27,28,29,30,31,32,33,34,35,36,37,38,39,40,41,42,43,44,45,46,47]; Table S5: Editing efficiencies of foundational prime editing systems in various plant species [9,13,14,15,16,17,19,20,25,28].

## Abbreviations

The following abbreviations are used in this manuscript:

*PE*	prime editing
*Cas9*	associated protein 9
*RT*	reverse transcriptase
*CRISPR/Cas9*	Clustered Regularly Interspaced Short Palindromic Repeats/associated protein 9
*DSBs*	DNA double-strand breaks
*NHEJ*	non-homologous end joining
*indels*	insertions or deletions
*HDR*	homology-directed repair
*BE*	base editing
*nCas9*	*Cas9* nickase
*pegRNA*	prime editing guide RNA
*PAM*	protospacer adjacent motif
*PRISMA*	Preferred reporting items for systematic reviews and meta-analyses
*NLS*	nuclear localization signal
*NC*	nucleocapsid
*mPE*	modular *PE*
*RTT*	reverse transcriptase template
*MMR*	mismatch repair
